# Safety Footwear Impact on Workers’ Gait and Foot Problems: A Comparative Study

**DOI:** 10.3390/clinpract14040120

**Published:** 2024-07-29

**Authors:** Alberto Arceri, Antonio Mazzotti, Sofia Gaia Liosi, Simone Ottavio Zielli, Elena Artioli, Laura Langone, Francesco Traina, Lorenzo Brognara, Cesare Faldini

**Affiliations:** 1 1st Orthopaedics and Traumatologic Clinic, IRCCS Istituto Ortopedico Rizzoli, 40136 Bologna, Italy; alberto.arceri@ior.it (A.A.); antonio.mazzotti@ior.it (A.M.); simoneottavio.zielli@ior.it (S.O.Z.); elena.artioli@ior.it (E.A.); laura.langon@ior.it (L.L.); cesare.faldini@ior.it (C.F.); 2 Department of Biomedical and Neuromotor Sciences (DIBINEM), Alma Mater Studiorum University of Bologna, 40123 Bologna, Italy; sofiagaia.liosi@studio.unibo.it (S.G.L.); francesco.traina@ior.it (F.T.); 3 Ortopedia, Traumatologia e Chirurgia Protesica e dei Reimpianti di Anca e Ginocchio, IRCCS Istituto Ortopedico Rizzoli, 40136 Bologna, Italy

**Keywords:** safety shoe, safety boot, inertial sensor, skin lesion, discomfort, podiatric symptoms, occupational health

## Abstract

Background: In this study, we hypothesized that safety footwear (SF) impacts gait patterns, potentially contributing to the podiatric symptoms reported by workers. The purpose of this work was to compare the gait analyses of workers wearing SF and sneakers using inertial sensors while also examining the occurrence of foot problems. Methods: A consecutive cohort of workers from different occupational sectors who wore SF during their work shifts were prospectively assessed through a gait analysis. The gait analysis was conducted under two conditions: first, while wearing SF, and second, while wearing sneakers. In both conditions, inertial sensors were used (Wiva^®^ MOB). Participants also underwent a podiatric physical examination to evaluate foot problems. Results: This study shows that SF resulted in a worsening gait pattern compared to sneakers in both genders. The impact was particularly pronounced in female participants, resulting in a significant decline in walking speed and cadence. Discomfort was reported by 83.3% of participants, with a higher prevalence in females (46.6% vs. 36.6%). The SF group exhibited an elevated prevalence of foot problems, with no significant gender variations. It seems that foot problems are more likely to occur when a foot deformity, such as flat or cavus foot or hallux valgus, is present. Conclusions: This study suggests that SF may contribute to the reported podiatric symptoms among workers. Certain footwear characteristics, including weight, mis-fit, and inadequate design, may be factors associated with footwear discomfort and adverse gait patterns, potentially leading to increased foot problems among workers.

## 1. Introduction

In the realm of occupational safety, the utilization of Personal Protective Equipment (PPE) is mandatory in environments with potential hazards, and safety footwear (SF) stands out as a crucial component in preventing workplace injuries. SF includes various types of boots and shoes worn for approximately 8 h per day, 5 days a week by high-risk-of-injury workers [1].

The importance of SF in injury prevention is underscored by safety regulations like EN ISO 20345:2022 [2] in Europe, ASTM F2413-18 [3] in the United States, and AS 2210.3/2019 [4] in Australia, which standardize SF features to ensure adequate safeguarding of workers. Various SF models exist, each obligated to adhere to basic safety regulations, encompassing features like the toe cap, anti-penetration plate, and slip-resistant sole. Specific SF designs conform to required standards based on workplace hazards and the tasks undertaken by individual workers [5].

However, some investigations have pointed to discomfort associated with SF, including issues related to excessive heat, inflexible soles, footwear weight, and pressure from the steel toe cap [6,7,8,9]. Recognizing the progression from discomfort to pain [10], studies indicate that discomfort not only induces new foot problems but also exacerbates existing ones, with prevalent issues like painful feet and skin lesions [6]. This discomfort has socio-economic implications, contributing to increased absenteeism and reduced health-related quality of life. Notably, 25% of workers abstain from wearing SF due to reported discomfort, despite its mandatory usage [6,9]. This reluctance emphasizes the urgent need to address SF-related discomfort, not solely for compliance but also for the overall well-being and productivity of workers.

Nevertheless, the existing studies often rely on surveys to assess wearer acceptance and foot problem rates, with few delving into an often-overlooked aspect in the scientific literature—the potential impact of SF on gait patterns, which could significantly influence foot health [1,11,12,13,14,15,16,17,18].

To evaluate a correlation between the daily use of SF and the reported podiatric symptoms of workers, a gait analysis was performed using inertial sensors. A comparison between workers walking in SF and sneakers was to carried out to investigate the perceived discomfort of the workers and the incidence of foot problems. Addressing SF-related problems is crucial for improving worker compliance, overall well-being, and productivity.

## 2. Materials and Methods

After obtaining institutional review board approval and written informed consent from all participants, a cohort of consecutive workers from different occupational sectors, who wore SF during their work shifts, were recruited prospectively and voluntarily to assess gait patterns between SF and sneakers using inertial sensors. Additionally, participants underwent a physical examination to assess foot problems. The experimental study period ran from June to December 2023. 

The protocol was approved by the ethical committee, and all patients provided informed consent. This study was conducted in accordance with the ethical principles outlined in the Declaration of Helsinki and with the Guidelines for Good Clinical Practice.

### 2.1. Patient Selection

Participants were required to be workers who regularly wore SF for most of their daily work, at least 3 h per day for a minimum of 4 days per week. The age range was set between 18 and 60 years to obtain a broad yet pertinent demographic, including only active workers.

Exclusion criteria were established to maintain the focus of the study and to ensure a well-defined and representative sample. Participants who were retired, currently unemployed, or had genetic, severe musculoskeletal, visual, or neuro-degenerative disorders or cognitive decline were excluded, as these conditions could affect task performance or introduce confounding variables. Furthermore, only participants without pre-existing injuries were deemed eligible. Additionally, participants with dorsiflexion limitation were excluded to avoid issues related to altered gait patterns or plantar pressure distribution, and discomfort potentially exacerbated by SF limitations. Ankle dorsiflexion is essential for raising the foot during the swing phase of gait, allowing for adequate ground clearance and preventing trips and falls. When dorsiflexion is limited, compensatory movements occur in other joints and a shorter step length. This limitation often causes increased pressure on the forefoot, as the heel may lift off the ground prematurely. Additionally, restricted dorsiflexion compromises the body’s ability to maintain stability during walking, thereby increasing the risk of falls. Activities requiring a full range of ankle motion, such as squatting, climbing stairs, or running, become challenging and painful with limited dorsiflexion [19,20].

### 2.2. Safety Footwear and Sneakers

The SF worn by the participants during the test had to meet the basic requirements of the European safety standards (EN ISO 20345:2022 [2]), including a crush-resistant steel toe cap, slip- and puncture-resistant sole, a closed heel zone, and heel cushioning. The weight of the work shoes ranged from 550 to 650 g per foot. 

The sneakers included in this study had similar general characteristics: a lightweight and flexible upper, a round and wide toe box, a cushioned and flexible sole, and a drop of at least 2 cm (the difference in height between the heel and forefoot areas of the midsole). The weight of the sneakers was between 250 and 300 g per foot.

### 2.3. Inertial Sensor Measuring

**Figure 1:**
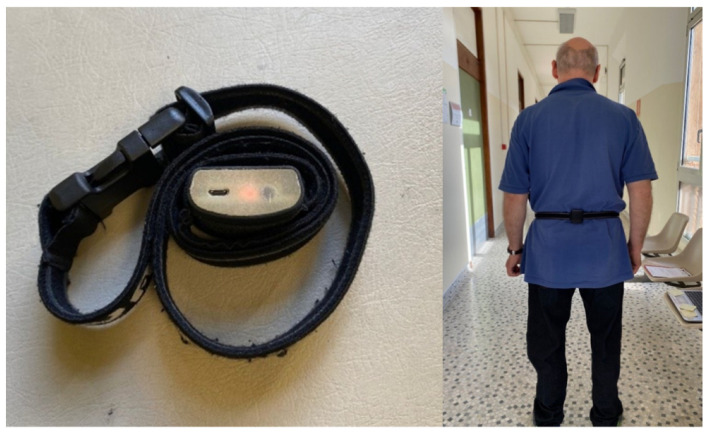
Wiva^®^ MOB device in use for kinematics analysis in this study (**left**); example of sensor positioning on a participant (**right**).

The kinematic analysis of walking was performed using the Wiva^®^ MOB device (Figure 1), which was specifically designed to measure gait characteristics. This device uses a system of inertial sensors, including a 9-axis Inertial Measurement Unit (IMU), an altimeter, and a GPS, recording at a sampling rate of up to 200 Hz. Light and compact (only 30 g), the device can be attached to the patient’s back with an ergonomic and adjustable belt, minimizing the impact on performance during the test. The sensor recorded signals during the test and transmitted the data to a computer via Bluetooth. The Biomech Studio 2018 software automatically generated a gait analysis report based on the sensor input, presenting spatiotemporal and specific kinematic parameters, including the following:

- Walking speed [m/min]: the distance covered by the patient in one minute of walking;
- Cadence [steps/min]: the number of steps per minute;
- Stride length [m]: the distance between two consecutive heel strikes of the same foot;
- Step duration [s]: the time between ipsilateral and contralateral heel strikes;
- Stance duration [%]: the foot support phase, from heel strike to toe-off of the same foot, typically about 62% of the gait cycle;
- Swing duration [%]: the foot swing phase, from toe-off to heel strike of the same foot, typically about 38%;
- Double support duration [%]: the duration of the stance phase on both feet as a per-centage of the gait cycle (approximately 20% of the stance phase).

### 2.4. Experimental Design

**Figure 2:**
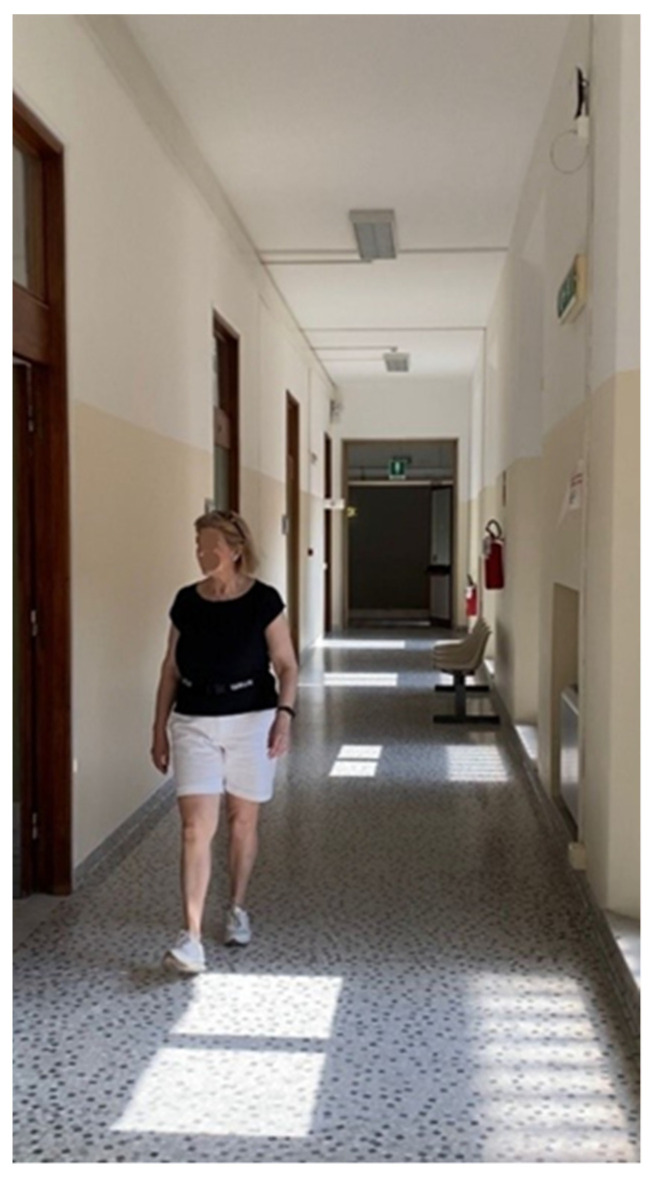
The 10 m walk test.

Once the Wiva^®^ MOB device was set up according to the manufacturer’s guidelines, all participants were verbally instructed on the test to be performed and given a practical example. The test involved walking on a flat path, following a linear trajectory for a predetermined length of 10 m (Figure 2). The experimental protocol consisted of two sequential 10 m walks under different conditions. First, each participant performed a 10 m walk while wearing SF. Then, each participant put on sneakers and performed a second 10 m walk. In both conditions, participants were asked to reproduce their natural posture, allowing for spontaneous and normal walking, keeping their head upright and arms by their sides. This study was carried out in accordance with the World Medical Association Declaration of Helsinki.

Kinematic gait analysis data were collected and stored by the recording device, transferred to the software, and manually tabulated on an Excel spreadsheet for each patient session.

### 2.5. Clinical Examination

All patients underwent a clinical examination by a podiatrist to determine the presence of work-related foot lesions, encompassing hyperkeratosis, corns, onycholysis, onychomadesis, onychomycosis, and subungual hematomas. A control group of 30 people who had never used SF and with homogeneous demographic characteristics to the SF group was compared to establish a more realistic prevalence of work-related skin lesions. Complaints of discomfort from participants were also investigated. Clinical data were recorded by a podiatrist who noted the presence of foot lesions and perceived discomfort on an assessment form using a dichotomous yes/no response.

### 2.6. Statistical Analysis

The mean values of each kinematic parameter were compared between the SF group and the sneaker group. Additionally, a gender comparison was performed.

Data collection was carried out utilizing Microsoft Excel 365 (Microsoft Corporation, Redmond, WA, USA) for Windows 11, and statistical analysis was performed using the software Jamovi project (2022) version 2.3.

Continuous variables were reported as mean values and standard deviations, while categorical variables were reported as percentages and frequencies. Differences in continuous variables were analyzed using Student’s *t*-test. Analysis was performed using chi-square testing when appropriate to determine differences in categorical variables between study groups. Statistical significance was set at a *p*-value less than 0.05, per standard convention.

## 3. Results

### 3.1. Population

This study recruited and analyzed 30 workers who regularly wore SF. Their characteristics are shown in Table 1. In addition, a control group of 30 subjects with similar demographic characteristics was selected for a clinical comparison of foot problems (Table 1).

### 3.2. Inertial Sensor Parameter Outcomes

Gait analysis using inertial sensors compared kinematic parameters between measurements taken with SF and sneakers. The results revealed a statistically significant decrease in speed, cadence, and stride length, while step duration and its subphases increased significantly when wearing SF (Table 2).

Within the gender subgroup analysis, the kinematic parameters again showed a statistically significant worsening in gait when wearing SF compared to sneakers for both the male and female groups (Table 2). In detail, males showed significant reductions in walking speed, cadence, and stance duration with SF compared to sneakers, whereas in females, a significant deterioration in all kinematic parameters was observed.

Notably, the observed differences in kinematic parameters between males and females when wearing SF was statistically significant for walking speed and cadence (Table 2).

### 3.3. Foot Problems

**Figure 3:**
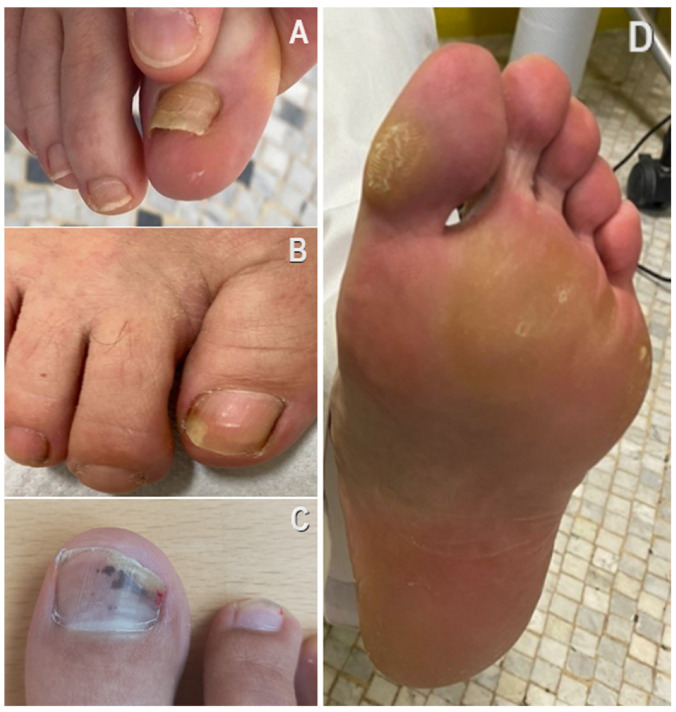
Onychomadesis and distal onycholysis of the hallux nail plate (**A**); distal onycholysis of the hallux lateral nail edge (**B**); subungual hematoma of the hallux nail plate (**C**); plantar corn and hyperkeratosis (**D**).

The physical examination conducted on the participants revealed the presence of several podiatric issues (Figure 3). The prevalence of each foot problem within the SF group was compared with the control group, revealing a statistically significant increase in foot problems’ occurrence for the SF group, except for plantar fasciitis (Table 3).

When considering gender subgroups, among participants wearing SF, a higher prevalence in females was found for hyperkeratosis, corns, onycholysis, onychomycosis, and subungual hematomas, but it did not reach statistical significance (Table 3).

Moreover, there was an incidence of six flat feet, two cavus feet, and thirteen hallux valgus cases in the SF group. In contrast, the control group exhibited four flat feet, three cavus feet, and seven hallux valgus cases. All patients (100%) with foot deformities (n = 15) in the SF group reported at least one foot problem, whereas only four out of nine (44%) individuals in the control group with foot deformity had a foot problem (*p*-value = 0.0014).

Discomfort was reported by 25 participants (83.3%), with a higher prevalence in females (46.6% vs. 36.6%). A considerable proportion of the respondents (92%) identified the heaviness of the SF as a discomfort factor, followed by excessive sweating (73.3%) and a sensation of pressure at the toe cap (60%).

## 4. Discussion

The purpose of the present study was to compare the gait patterns of workers wearing SF and sneakers using inertial sensors while also examining the occurrence of foot skin lesions. 

The results showed that SF resulted in a worsening gait pattern compared to sneakers for both genders. However, the effect was more pronounced in the female participants, indicating a significant deterioration in walking speed and cadence. In addition, the prevalence of foot problems increased in the SF group, although no significant gender differences were found. Workers wearing SF exhibit an altered gait pattern that can be correlated with findings reported in various studies in the literature. Previous research has demonstrated that SF can affect the stance phase, notably impairing step time, step length, and stride length [18,21]. Furthermore, there is evidence of altered lower limb muscle activity, presumably due to attempts to compensate during the gait cycle, resulting in increased energy expenditure [14], affecting oxygen consumption [15] and metabolic indicators [13], all indicative of increased muscle fatigue. Thus, wearing SF adversely impacts gait and increases muscle fatigue, which is recognized as a precursor to musculoskeletal disorders such as lower limb pain [16,17,22]. Moreover, the altered gait is reflected in an abnormal plantar pressure distribution [11], particularly an increase in pressure–time integrals, a critical factor in ulcer formation [23]. This abnormal gait pattern with SF may lead to foot conflict with the shoe, resulting in injuries due to increased friction and mechanical pressure, including blisters, hyperkeratosis, corns, onycholysis, and subungual hematomas [24]. These considerations are supported by the higher prevalence of foot problems observed in our findings in the SF group.

Furthermore, the causes of the altered gait pattern could be attributed to factors associated with footwear discomfort, including heaviness, mis-fitting, and inadequate design. Many studies report the weight of the SF as a factor commonly complained about by workers [6,7,11]. The weight of the SF, typically between 0.9 and 1.5 kg, up to 4.4 kg in certain cases [13,25], is consistent with the weight of the footwear selected in this study. The significant weight is mainly due to the steel toe-cap and anti-penetration plate, but materials, sole type, and shaft height also play a role [11].

Mis-fitting footwear, either too small or too wide, prevents the foot from stabilizing internally, increasing stress on foot structures during walking. Tight-fitting shoes often cause discomfort and tissue compression damage, while loose footwear may lead to friction-related issues [26]. Similarly, foot sweating can not only lead to prolonged exposure to moisture [6,9] but also inadequate grip inside the footwear, resulting in rubbing between the foot and the shoe.

The SF design, particularly ankle support, was scrutinized and criticized in many studies [27,28]. Different SF designs must always be contextualized to task performance and the working environment in which the user operates. For instance, a stiff boot shaft may limit ankle movement [29], especially for workers on tilted surfaces [28] or requiring compensatory foot adjustments for stability on uneven surfaces [27].

The comparison between males and females revealed that the female gender experienced a greater impairment in gait when wearing SF, as evidenced by a statistically significant deterioration in kinematic parameters during gait analysis, particularly in walking speed and cadence, compared to their male counterparts. Similarly, the prevalence of foot skin lesions was higher in females than in males, although this did not reach statistical significance in this limited population sample.

Females are less tolerant and less accepting of SF than males [8], with males more likely to wear them (80%) than females (60%) [6]. Extensive research has also confirmed the morphological differences between male and female feet, demonstrating that female feet and legs are not simply reduced versions of male feet and legs, but have significantly different morphologies [30,31]. It is therefore clear that women’s footwear should not be made as a reduced version of men’s footwear in order to achieve an appropriate fit.

Considering foot deformities, it appears that foot problems are more likely to occur in SF when a foot deformity is present. To our knowledge, this is the first study that examines pre-existing foot deformities in relation to the foot problems identified with the use of SF. The results suggest that in the presence of foot deformities is associated with a higher risk of developing skin lesions. This is likely to be related to a more pronounced mis-fit for these feet, leading to increased friction and pressure, causing conflicts with SF. A customized approach in the presence of these deformities is thus recommended.

Based on these findings, several recommendations are proposed to address reticence to SF use. These include exploring innovative lightweight materials to reduce the weight of the footwear, considering variations in foot shapes, and implementing different shoe sizing for an optimal fit. Additionally, assessing the flexibility of the shaft/sole and providing ankle support tailored to the task activities and the working environment are crucial. It is also advisable to incorporate proper podiatric counselling for wearers to proactively prevent foot-related problems.

This study has limitations, including the small size of the patient sample, although a rigorous patient selection process was used to exclude conditions that could affect gait analysis and introduce potential confounders. 

Additionally, it is important to note that discomfort is a self-reported outcome, lacking objective measurability. Further studies should also include objective measures of discomfort to improve interpretation of the data. Relying solely on subjective reports may not accurately capture the level and nature of discomfort experienced by workers. Objective measures such as pressure mapping, gait analysis, and biomechanical assessments can provide quantifiable data to improve our understanding of how SF affects foot health. 

Another limitation was the lack of joint flexibility/laxity assessment, thus missing potential data on joint hypermobility, which may impact the comprehensiveness of our findings regarding its association with SF. In particular, joint laxity exceeds the normal range of motion, potentially influencing postural balance and lower extremity alignment, which may alter gait dynamics. Current knowledge on pedobarographic differences in joint laxity is limited and often focuses on foot-type deviations [32,33]. Some studies have been carried out, for example, on differences in foot pressure measurements between men and women [34,35], or in Ehlers–Danlos syndrome, which shows significant differences in plantar pressure but is confounded by additional symptoms such as neurological dysfunction and pain [33]. No reliable evidence exists on how varying degrees of hypermobility impact plantar loading patterns [36]. Further studies on the lower limb biomechanics in individuals with joint laxity can enhance our understanding of the effects of laxity on gait, including in different settings.

## 5. Conclusions

Current SF, while designed to meet safety standards, often leads to foot problems and frequent discomfort. This study has shown that wearing SF led to a deteriorated gait pattern compared to sneakers for both genders, with a more significant impact on females. The SF group exhibited a high prevalence of foot problems, although no significant gender differences were found. Foot deformities also increased the likelihood of foot problems when wearing SF. These findings indicate that SF may contribute to the reported podiatric symptoms due to work-related foot lesions. Footwear characteristics, such as weight, mis-fit, and inadequate design, may be factors associated with footwear discomfort and may potentially explain the observed alterations in gait pattern and foot problems. Further research efforts are needed to improve SF and raise awareness among professionals of the need to consider gender differences and pre-existing foot deformities in workers using SF looking for a personalized approach.

## Figures and Tables

**Table 1:** Patients’ characteristics.

	SF Group	Clinical Control Group	*p*-Value
**Patients**	30	30	
**Age**	50.4 ± 9.5 (CI 95% = 46.7–54.1)	46.9 ± 10.7 (CI 95% = 42.7–51.1)	*0.179*
**Gender**	14M/16F	15M/15F	
**BMI**	20.8 ± 3.6 (CI 95% = 19.4–22.2)	22.4 ± 3.7 (CI 95% = 20.9–23.8)	*0.091*

Abbreviations: **M** male; **F** female; **BMI** body mass index.

**Table 2:** Inertial sensor parameter outcomes.

	Walking Speed (m/min)	Cadence (steps/min)	Stride Length (m)	Step Duration (s)	Stance Duration (%)	Swing Duration (%)	Double Support Duration (%)
**SF**	79.2 ± 20.8 (CI 95% = 71.2–87.3)	79.1 ± 15.5 (CI 95% = 73.1–85.1)	0.7 ± 0.4 (CI 95% = 0.6–0.9)	0.9 ± 0.4 (CI 95% = 0.8–1.1)	61.6 ± 2.3 (CI 95% = 60.8–62.5)	37.6 ± 3.4 (CI 95% = 36.3–39.0)	12.4 ± 2.3 (CI 95% = 10.2–12.4)
**Sneakers**	66.2 ± 15.9 (CI 95% = 60.1–72.4)	68.7 ± 17.8 (CI 95% = 61.8–75.7)	0.9 ± 0.1 (CI 95% = 0.9–1.0)	0.8 ± 0.2 (CI 95% = 0.7–0.9)	60.4 ± 2.6 (CI 95% = 59.4–61.4)	35.9 ± 2.5 (CI 95% = 34.9–36.9)	11.3 ± 2.9 (CI 95% = 11.5–13.3)
***p*** **-value**	***<0.001***	***<0.001***	***0.008***	***0.059***	***<0.001***	***0.020***	***0.034***
**SF male**	68.3 ± 12.1 (CI 95% = 60.6–76.0)	70.6 ± 8.5 (CI 95% = 65.2–76.1)	0.8 ± 0.2 (CI 95% = 0.7–1.0)	1.1 ± 0.5 (CI 95% = 0.8–1.5)	61.9 ± 1.8 (CI 95% = 60.7–63.1)	39.1 ± 3.4 (CI 95% = 36.9–41.3)	11.5 ± 2.0 (CI 95% = 8.6–11.7)
**Sneakers male**	56.9 ± 16.6 (CI 95% = 46.4–67.5)	56.9 ± 19.1 (CI 95% = 45.5–68.5)	0.9 ± 0.1 (CI 95% = 0.9–1.0)	0.9 ± 0.2 (CI 95% = 0.8–1.0)	59.9 ± 2.8 (CI 95% = 58.1–61.7)	36.6 ± 2.5 (CI 95% = 35.0–38.3)	10.2 ± 2.4 (CI 95% = 10.2–12.7)
***p*** **-value**	***0.011***	***0.012***	*0.331*	*0.239*	***<0.001***	*0.122*	*0.199*
**SF female**	87.5 ± 22.4 (CI 95% = 75.5–99.4)	85.4 ± 16.8 (CI 95% = 76.2–94.4)	0.6 ± 0.4 (CI 95% = 0.4–0.8)	0.8 ± 0.1 (CI 95% = 0.7–0.8)	61.4 ± 2.6 (CI 95% = 60.1–62.8)	36.6 ± 3.0 (CI 95% = 35.0–38.2)	13.1 ± 2.3 (CI 95% = 10.6–13.7)
**Sneakers female**	73.2 ± 11.6 (CI 95% = 67.0–79.4)	77.6 ± 11.8 (CI 95% = 71.3–83.9)	0.9 ± 0.1 (CI 95% = 0.8–1.0)	0.7 ± 0.1 (CI 95% = 0.6–0.8)	60.7 ± 2.4 (CI 95% = 59.4–62.0)	35.4 ± 2.5 (CI 95% = 34.1–36.7)	12.1 ± 2.9 (CI 95% = 11.8–14.3)
***p*** **-value**	***0.002***	***0.015***	***0.013***	***0.002***	***0.014***	***0.050***	***0.074***
***p*** **-value** **SF M/F**	***0.015***	***0.019***	*0.232*	*0.068*	*0.439*	*0.076*	*0.195*

Abbreviations: **SF**, safety footwear. **Bold** *p*-value indicates reaching statistical significance. NORMAL VALUES walking speed: 77.4 ± 9.4 m/min for male, 71.4 ± 10.2 m/min for female; cadence: 52.8 ± 3.8 steps/min for male, 55.8 ± 4.4 steps/min for female; stride length: 1.46 ± 0.13 m for male, 1.28 ± 0.15 m for female; step duration: 1.14 ± 0.08 s for male, 1.08 ± 0.08 s for female; stance duration: 60.3 ± 1.7%; swing duration: 39.6 ± 1.9%; double support duration: 9.4 ± 2.3% for male, 9.6 ± 4.6% for female.

**Table 3:** Foot problems’ prevalence.

	*Hyperkeratosis*	Corns	Onycholysis/Onychomadesis	Onychomycosis	Subungual Hematomas	*Plantar fasciitis*
***SF total (30)***	28 (93.3%)	17 (56.6%)	9 (30%)	6 (20%)	22 (73.3%)	5 (16.7%)
***CG (30)***	9 (30%)	3 (10%)	1 (3.3%)	0	2 (6.7%)	1 (3.3%)
***p*** **-value**	***<0.0001***	***<0.0001***	***0.005***	***0.010***	***<0.0001***	*0.086*
**SF male (14)**	12 (85.7%)	6 (42.8%)	3 (21.4%)	2 (14.3%)	10 (71.4%)	3 (21.4%)
**SF female (16)**	16 (100%)	11 (68.7%)	6 (37.5%)	4 (25%)	12 (75%)	2 (12.5%)
***p* -value**	*0.124*	0.160	0.345	0.472	0.885	0.521

Abbreviation: **SF,** safety footwear. **Bold** *p*-value indicates reaching statistical significance. The different coloring was planned to differentiate the values reported by the male group and the female group in order to clarify the interpretation.

## Data Availability

The data presented in this study are available on request from the corresponding author. The data are not publicly available due to privacy.
